# Social Inequalities in Changes in Diet in Adolescents during Confinement Due to COVID-19 in Spain: The DESKcohort Project

**DOI:** 10.3390/nu13051577

**Published:** 2021-05-08

**Authors:** Alicia Aguilar-Martínez, Marina Bosque-Prous, Helena González-Casals, Ester Colillas-Malet, Susanna Puigcorbé, Laura Esquius, Albert Espelt

**Affiliations:** 1 Foodlab Research Group, Faculty of Health Sciences, Universitat Oberta de Catalunya, Rambla del Poblenou, 156, 08018 Barcelona, Spain; aaguilarmart@uoc.edu (A.A.-M.); lesquius@uoc.edu (L.E.); 2 Faculty of Health Sciences, Universitat Oberta de Catalunya, Rambla del Poblenou, 156, 08018 Barcelona, Spain; 3 Departament de Psicobiologia i Metodologia en Ciències de la Salut, Universitat Autònoma de Barcelona (UAB), C/ de Ca n’Altayó s/n, 08193 Bellaterra, Spain; aespelt@umanresa.cat; 4 Department of Epidemiology and Public Health, Faculty of Health Sciences of Manresa, Universitat de Vic–Universitat Central de Catalunya (UVic-UCC), Av. Universitària 4-6, 08242 Manresa, Spain; HGonzalez@umanresa.cat (H.G.-C.); ecolillas@umanresa.cat (E.C.-M.); 5 Program on Substance Abuse, Public Health Agency of Catalonia. C/ Roc Boronat, 81–95, 08005 Barcelona, Spain; susanna.puigcorbe@gencat.cat; 6 Centro de Investigación Biomédica en Red de Epidemiología y Salud Pública (CIBERESP), C/ Monforte de Lemos 3 Pabellón 11, 28029 Madrid, Spain

**Keywords:** adolescent, food habits, socioeconomic position, social inequalities, COVID-19

## Abstract

Adolescence is a critical period in the consolidation of healthy lifestyles that can last into adulthood. To analyze changes in food consumption and eating behaviors in high-school adolescents during the first confinement, a cross-sectional study was conducted at the end of confinement in Spain. Changes in the frequency or quantity of consumption of different types of food and food-related behaviors were analyzed. Socioeconomic and health-related variables were also considered. To determine whether dietary changes were related to socioeconomic position (SEP), Poisson regression models with robust variance were estimated. Overall, there were some changes towards a healthier diet such as an increase in fruit consumption (38.9%) and a decrease in the consumption of soft drinks (49.8%), sweets and pastries (39.3%), and convenience foods (49.2%). Some changes, however, were related to less healthy behaviors, such as a more irregular pattern of meal distribution (39.9%) or an increase in snacking between meals (56.4%). Changes towards less healthy eating were also related to students’ SEP. The risk of worsening the diet was found to be 21% higher in adolescents from a more disadvantaged SEP. Future public policies could be adapted to avoid increasing nutritional and health inequalities.

## 1. Introduction

The outbreak of the coronavirus disease 2019 pandemic, COVID-19, caused a global crisis with a major impact on health, but also on society and the economy. It forced most countries to establish containment measures to control the overload of health services and reduce the spread of the virus [1].

In the case of Spain, the expansion of the disease forced the country to establish a state of alarm in mid-March and to adopt significant measures restricting mobility and ordinary activities. Such measures consisted of a long period of total lockdown [2], which, while necessary to minimize the rate of infection, involved lifestyle changes that also negatively impacted on health. Some of these negative health effects have been increased sedentarism; decreased physical activity; changes in emotional state (stress, anxiety, depression, etc.); altered nutritional status (linked to energy imbalance or nutritional composition of the diet in macro- or micro-nutrients) [3,4,5,6].

Adolescence is a critical period to consolidate healthy lifestyles that can last into adulthood. Knowing the food habits of adolescents and the determining factors is of great importance. Indeed, these habits can have an impact on people in a vital moment of their development, such as adolescence, but also in the future [7]. Adolescence is also a time when relationships with family, peers, and school play a crucial role and must be considered among the determinants of health [8].

Adequate food habits and a healthy diet are important factors in maintaining health and preventing diseases, such as obesity, diabetes, cardiovascular problems, and cancer [9]. However, in recent years, different studies have shown a progressive loss, especially among adolescents and young people, of healthy eating patterns, such as the Mediterranean Diet [10,11]. These studies have also shown a lower than recommended consumption of fruit, vegetables, and dairy products and a higher than recommended consumption of meat and meat products, fats, and sweets [12].

For adolescents, confinement caused important changes at different levels: physical activity (no more traveling to and from school, playtime, sports activities, etc.); eating habits (all the meals of the day at home, in most cases with the family); social relations (less physical interaction between peers, greater coexistence with the family, etc.) [13]. Some studies have shown that the possibility of having more home-cooked meals with the family could contribute to improving the nutritional habits of adolescents [14,15]. However, other studies have demonstrated that increased free time in front of screens or boredom could lead to excessive consumption of highly energetic food and food of poor nutritional quality [16,17]. Besides, there is a known relationship between unhealthy habits and a more disadvantaged socioeconomic position [18,19]. Therefore, it is also important to study the effects of adopting the same measures for everyone during confinement, as this could lead to increase nutritional inequalities.

Therefore, knowing how confinement has affected the quality of the diet of adolescents could help to understand which factors can have a more decisive influence on their eating behavior. This knowledge will help to design policies aimed at improving lifestyles during adolescence and have an impact not only on current health, but also on the prevention of diseases in the adult stage. For this reason, the main aim of this study was to analyze changes in food consumption and eating behaviors in high-school adolescents during the first lockdown (March–May 2020). We also wanted to ascertain whether these changes vary according to the quality of the students’ diet before the COVID-19 pandemic and to their perceived socioeconomic position.

## 2. Materials and Methods

This study was part of the DESKcohort project [20], which monitors 12- to 18-year-old students that attend educational centers in Central Catalonia over time. The aim was to analyze their behavior or relevant aspects that could affect their health and their social and educational life.

To conduct this study, we used a cross-sectional design, combining data from two parts of the DESKcohort project: the first one conducted before the COVID-19 pandemic (DESKcohort project first wave from October 2019 to February 2020) and the second one conducted at the end of the confinement in Spain (DESK-COVID project from June to July 2020). The study population consisted of 303 14- to 18-year-old students from a secondary school in Central Catalonia during the academic year 2019–2020. These students had participated in the first wave of the DESKcohort survey and agreed to be contacted for further studies. The DESKcohort questionnaire was self-administered in high schools using a tablet, whereas the DESK-COVID survey was sent by email or WhatsApp to the students. This second survey was a reduced version of the first one, including questions related to the effects of COVID-19 pandemic. Both questionnaires included data on health and health-related behaviors. Previous responses of demographic and socioeconomic factors (DESKcohort questionnaire) were merged into the data of the second questionnaire (DESK-COVID survey).

Information on food consumption prior to the COVID-19 pandemic confinement was collected through a food frequency questionnaire, which allowed us to estimate the degree of quality of each participant’s diet using the Spanish adaptation of the Healthy Eating Index, made by Norte-Navarro and Ortiz-Moncada in 2011 [21]. Based on the score obtained (range 0–100), the quality of the participants’ diets was divided into three categories: unhealthy diet (<50 points); diet needs changes (50–80 points); healthy diet (>80 points).

To determine whether changes in diet and eating behaviors had occurred during the COVID-19 pandemic confinement, for each variable we asked whether the participant’s frequency of consumption or behavior had decreased, remained unchanged, or increased compared to the pre-pandemic period. In total, we analyzed 18 variables on types of food and eating behaviors. The variables related to the consumption of different types of food were the following: (1) cereals; (2) legumes; (3) fruit; (4) vegetables; (5) dairy products; (6) meat; (7) fish; (8) eggs; (9) convenience food; (10) sweets and pastries; (11) soft drinks. The variables also included different eating behaviors related to: (12) the amount of food consumed; (13) variety of foods consumed; (14) regularity of meal hours; (15) meals skipped; (16) consumption of snacks between meals; (17) consumption of fresh food; (18) consumption of packaged food. Each variable had 3 response options: decrease in quantity or frequency; no change in quantity or frequency; increase in quantity or frequency.

Besides these variables, we also considered the following demographic, socioeconomic, and health-related factors: (a) gender (girl, boy); (b) course (ISCED 2 and ISCED 3, according to the UNESCO International Standard Classification of Education) [22]; (c) perceived socioeconomic position, which was a continuous variable adapted from the MacArthur Scale of Subjective Social Status [23]. To create the variable, we used the following question: “Now assume that the bar represents the position of people according to their neighborhood. At the top of the bar are the people who are in the highest position in the neighborhood. At the bottom are the people in the lowest position. Considering the standard of living of the people in your neighborhood, where would you place yourself on this scale?”. Values ranged from 0 to 100. To facilitate subsequent interpretation of the results, we recoded the variable so that higher values indicated a more disadvantaged socioeconomic position, while lower values represented a more advantaged socioeconomic position; (d) size of municipality (≤5000 inhabitants; 5001–20,000 inhabitants; >20,000 inhabitants); (e) body mass index (BMI) was defined using age- and sex-specific BMI cut-offs [24]. Adolescents’ heights and weights were self-reported. Given that only 7 participants were classified as underweight, the variable was dichotomized in (1) underweight or normal weight or (2) overweight or obesity; (f) physical activity, which was estimated from the average daily minutes of moderate or vigorous physical activity reported by the adolescents. Participants were divided into 2 groups according to whether they reached the WHO recommendations for physical activity in adolescents (>60 min per day) or not [25]; (g) self-perceived health, which was created from the question: “Would you say that in general your health is?”, with 5 response options (excellent, very good, good, fair, and poor). As there were only 6 participants who reported poor health status, the responses were recoded into 2 categories: (1) excellent or very good and (2) good, fair or poor; (h) sleep quality, which was a self-reported variable built from the question: “During the last month, how would you rate, in general, the quality of your sleep?”. The response categories were classified in two groups: (1) very good or good and (2) poor or very poor.

### Data Analysis

First, we described the main characteristics of the participants. Then, for each of the 18 eating variables, we estimated the proportion of students who increased, maintained, or decreased the quantity of their food intake or the frequency of their food-related behaviors during the COVID-19 confinement. Proportions were compared using Pearson’s chi2 test and Fisher’s exact test, and 95% confidence intervals (95% CI) were calculated.

Second, we wanted to further analyze whether changes in food consumption and food-related behaviors had occurred differently between participants, depending on how healthy the previous diet was. Therefore, we estimated a Healthy Eating Index (HEI). Afterwards, we divided the sample in tertiles according to the students’ score in the HEI. We finally calculated the percentage of participants showing a change for each of the 18 eating variables in each tertile. To ascertain whether there were significant differences in the changes in each of the variables, Pearson’s chi2 test was used, and 95% CI were calculated.

Finally, we determined if there were differential changes according to the students’ perceived socioeconomic position, for each of the 18 eating variables. To this aim, we dichotomized each variable to compare the least healthy category to the other two (e.g., decreased consumption of a healthy type of food versus same or increased consumption). Afterwards, we used the Poisson regression models with robust variance and their corresponding 95% CI [26,27], adjusting for the demographic, socioeconomic, and health-related variables. The crude and adjusted prevalence ratios (PR) were obtained by dividing the prevalence of a decrease in the intake or behavior over the prevalence of its maintenance or increase, or vice versa (i.e., increase vs. maintenance or decrease) (depending on the least healthy category in each variable). The final adjusted Poisson regression models included only the statistically significant variables. In addition, we calculated the pooled mean of the associations considering the point estimate and the effect size. All statistical analyses were conducted with STATA 16.

## 3. Results

Table 1 shows the baseline characteristics of the study sample. The mean age of the study population was 16.4 years (SD = 1.11), and around 70% of participants were girls. The mean perceived socioeconomic position was 62.5 (SD = 13.8), in a scale from 0 to 100. In the overall sample, we could distinguish different populations: 28.7% of participants lived in a municipality of less than 5000 inhabitants; 41.6% in a municipality of 5001 to 20,000 inhabitants; 26.1% in a municipality of more than 20,000 inhabitants; 3.6% without available data. Moreover, we collected the following health-related data: 82.5% of the people presented normal weight or underweight; 59.7% did not meet WHO recommendations on physical activity; 53.8% reported excellent or very good self-perceived health status; 60.7% considered they had very good or good sleep quality.

The overall changes in consumption of different food types and eating behaviors during confinement due to the COVID-19 pandemic are shown in Supplementary Figure S1. Around 40% of participants reported an increase in the consumption of fruit. Conversely, in the whole sample, consumption of sweets and pastries, convenience foods, and soft drinks decreased by 39.3, 49.2, and 49.8%, respectively. In relation to food-related behaviors, an increase in the variety of foods has been reported by 21.5% of the participants. In contrast, 56.4% of adolescents reported eating snacks between meals more frequently, 39.9% reported less regularity in meals distribution, and more than half reported changes in the number of meals (28.4% increased the number of meals versus 22.4% who decreased them).

**Figure 1:**
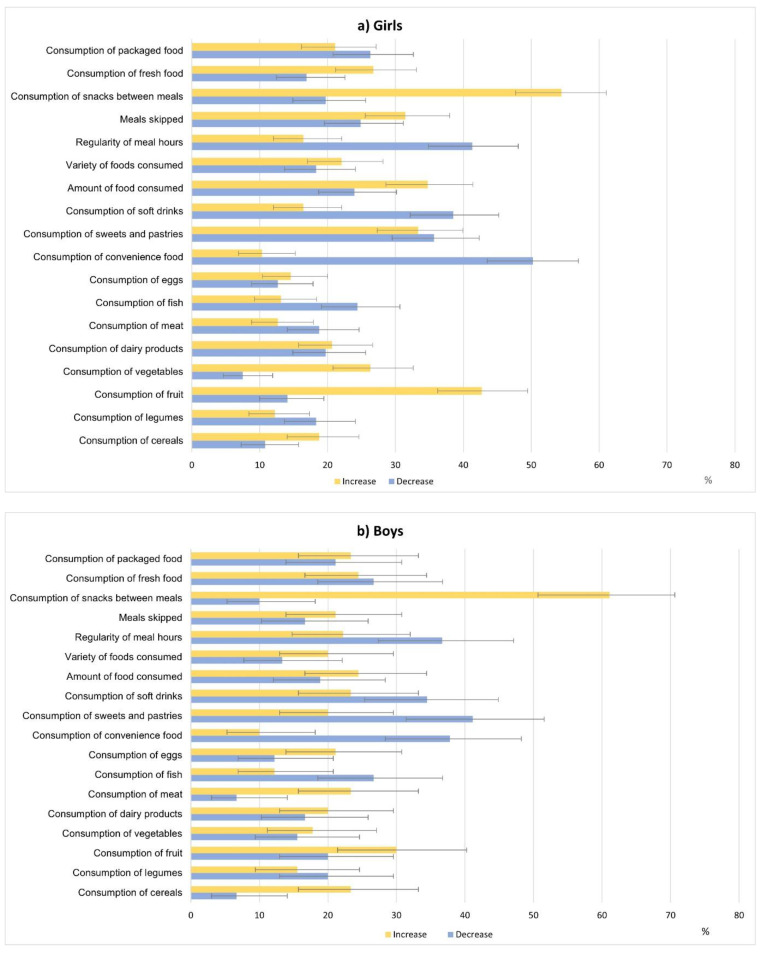
Changes in food consumption and eating behavior among high-school students in Central Catalonia during the confinement due to the COVID-19 pandemic. (**a**) Changes in girls; (**b**) Changes in boys.

The changes in consumption of different types of foods and food-related behaviors during the confinement due to the COVID-19 pandemic are shown in Figure 1a for girls and 1b for boys. For each variable of food intake or behavior, we compared the proportion of students who reported an increase to those who reported a decrease. In both girls and boys, eating snacks between meals was the behavioral variable that increased the most during the confinement [54.5% (95% CI = 47.7–61.1%) and 61.1% (95% CI = 50.7–70.6%), respectively]. The behavioral variable that decreased the most was the regularity of meal hours [41.3% (95% CI = 34.9–48.1%) and 36.7% (95% CI = 27.3–47.1%), for girls and boys, respectively]. Regarding food intake, we observed the highest increase in the consumption of fruits [42.7% (95% CI = 36.2–49.5%) in girls, and 30.0% (95% CI = 21.4–40.3%) in boys]. Moreover, among girls, we showed the highest decrease in the intake of convenience food [50.2% (95% CI = 43.5–56.9%)]. In boys instead, the consumption of sweets was the variable that decreased the most [41.1% (95% CI = 31.4–51.6)].

The average HEI score of the participants in the study, prior to confinement, was 68 points. We found statistically significant differences in the HEI prevalence before the COVID-19 confinement according to sex. In girls, 12.2% had a healthy diet, 79.8% needed some changes in their diet, and 8% had an unhealthy diet; whereas in boys, these proportions were 2.2, 93.3, and 4.4%, respectively (*p*-value < 0.01). No statistically significant differences were observed for the other variables studied. The proportion of participants showing changes in the consumption of different types of food and in eating behaviors during the COVID-19 confinement is shown in Table 2 and Table 3, respectively. Results are reported by tertiles of the Spanish Health Eating Index (HEI). There are no statistically significant differences in the consumption of any type of food, nor in any eating behavior, according to the dietary quality prior to COVID-19 confinement.

**Figure 2:**
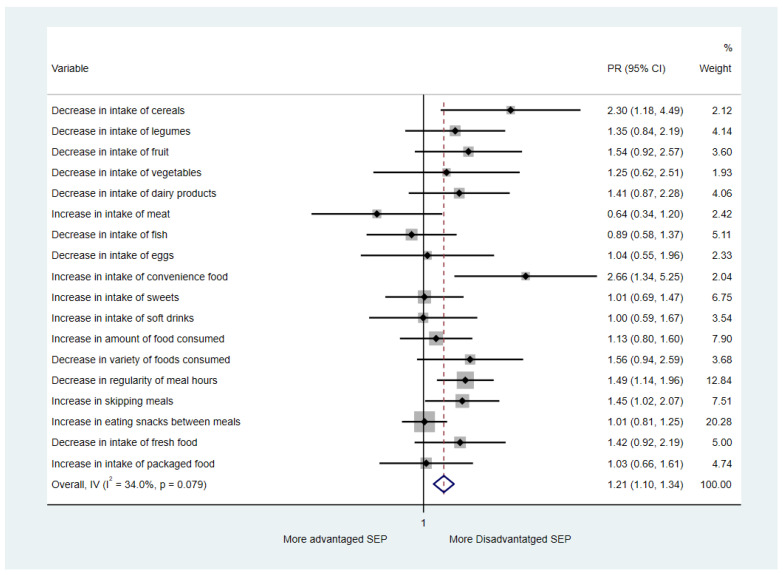
Relationship between less healthy eating variables during the COVID-19 confinement and a more disadvantaged socioeconomic position of the high-school students from Central Catalonia. Perceived socioeconomic position has been considered as a dichotomous variable: the most disadvantaged tertile versus the medium and more advantaged tertiles. Prevalence ratio has been adjusted by sex, age, and self-perceived health status. Abbreviations: PR: Prevalence Ratio; SEP: Socioeconomic Position; IV: Independent Variable; I^2^: I-squared (proportion of total variation in effect estimate due to between-study heterogeneity).

Poisson regression models adjusted by sex, age, and self-perceived health status were fit to ascertain whether there were differential changes in food intake and food-related behaviors depending on the students’ perceived socioeconomic position (continuous variable). Their results are presented in Supplementary Figure S2. In relation to food intake, we found that the reduction in consumption of cereals (PR = 1.03; 95% CI = 1.00–1.05; *p*-value < 0.03), fruit (PR = 1.02; 95% CI = 1.01–1.04; *p*-value < 0.01), and vegetables (PR = 1.02; 95% CI = 1.00–1.04; *p*-value < 0.05) was higher in adolescents from a more disadvantaged perceived socioeconomic position. In contrast, the increase in the intake of convenience food was significantly higher among students from a more disadvantaged socioeconomic position (PR = 1.04; 95% CI = 1.01–1.06; *p*-value < 0.01). Besides, in relation to eating behaviors, we found a statistically significant higher reduction in the regularity of meal hours in more disadvantaged socioeconomic positions (PR = 1.01; 95% CI = 1.00–1.02; *p*-value < 0.01). Moreover, a statistically significant higher increase in skipping meals was shown among more disadvantaged socioeconomic positions (PR = 1.02; 95% CI = 1.00–1.03; *p*-value < 0.02). Overall, in the pool analysis, we found that for each one-point increase in the student’s perceived socioeconomic position (higher values indicate a more disadvantaged socioeconomic position), the risk to worsen their diet increased by 1%. As a sensitivity analysis, we divided the students’ perceived socioeconomic position in tertiles and applied several adjusted Poisson regression models. We compared the changes of the 18 eating variables in the disadvantaged socioeconomic position tertile with respect to the other two (Figure 2). Overall, in the pool analysis, we found that the risk to worsen their diet was 21% higher (PR = 1.21; 95% CI = 1.10–1.34) in adolescents of the disadvantaged socioeconomic position tertile, compared to the ones of the intermediate and advantaged tertiles.

## 4. Discussion

Confinement due to the COVID-19 pandemic has meant some changes towards a healthier diet, such as an increase in fruit consumption, in high-school students from Central Catalonia. However, other changes were related to less healthy behaviors, such as increased snacking between meals. When we compared changes in the diet and dietary patterns according to the quality of the diet before the COVID-19 pandemic, we found no statistically significant differences between groups. Nevertheless, we found that changes in diet and dietary patterns have been unevenly distributed by the perceived socioeconomic position. Students from the most disadvantaged socioeconomic tertile showed a 21% increased risk to worsen their diet and dietary patterns, compared to students from the medium and highest tertiles.

### 4.1. Changes in Food Consumption and Eating Behaviours during the COVID-19 Confinement

Of participants, 38.9% reported an increase in the consumption of fruit (higher proportion for girls). On the contrary, the consumption of sweets and pastries, convenience food, and soft drinks decreased by 39.3, 49.2, and 49.8%, respectively. The reduction in the intake of these types of foods tended to be higher in girls, except for sweets and pastries. Excluding the negative trend in fish consumption, these results are in line with those found in the Spanish general population during the confinement: a reduction in the intake of pastries, chocolate, and soft drinks and an increase in fruit intake [28]. The decrease in the consumption of soft drinks and sweets could be partly related to the decrease in social interaction with peers, since the two behaviors are highly associated [29,30,31]. The decrease in the consumption of convenience foods correlates with the reduction in fast-food intake observed in adolescents by Ruiz-Roso et al. [32]. This also coincides with the increase in the habit of cooking at home observed in different studies, and with the initial closure of fast-food or take-away establishments [33]. The healthy habits acquired, however, may not be sustained over time with the reopening of restaurants and reactivation of delivery services [33,34]. Moreover, an increase in the variety of foods has been reported by 21.5% of the participants, and it should also be consolidated with the return to “normality”. However, the general increase in the amount of food consumed and irregular patterns of meal distribution could lead to poor body weight control. Indeed, 56% of adolescents have been eating more snacks between meals, 39.9% have shown less regularity in meals distribution, and more than half have reported changes in the number of meals. These latter results also coincide with previous studies showing that boredom, stress, and screen time could lead to increased meal irregularity or higher energy intake [16,17].

### 4.2. Changes in Diet and Dietary Patterns according to the Quality of the Diet before the COVID-19 Pandemic

Our findings show that pre-confinement diet of most participants of the study needed some changes, which were related to lower than recommended consumption of fruit and vegetables and higher consumption of processed meats, pastries, soft drinks, and snacks. The proportion of participants who scored “eating healthy” in the Spanish version of the HEI was higher among girls. These results are in line with the observations of the Norte-Navarro study [21] on the quality of the diet. The latter showed that women and older people have a healthier diet. Our data are also in agreement with studies showing, in recent years, a progressive loss of healthy eating patterns, such as the Mediterranean Diet (abundance of plant-based foods, priority of fresh foods over convenience foods, moderate meat consumption, etc.), especially among adolescents and young people [10,11].

No statistically significant differences were found in food consumption, frequency, and eating behaviors, independently of the diet quality index prior to the COVID-19 pandemic. Nevertheless, students in the highest tertile of the healthy eating index had the greatest percentages of decline in soft drink consumption and the greatest percentages of increase in fruit consumption. On the contrary, students in the lowest tertile reported the smallest decreases in soft drink and convenience food consumption, and the smallest increases in fruit consumption. Students in the lowest third also reported greater increases in the amount of food consumed, greater decrease in regularity in schedules, and greater decreases in the variety and consumption of fresh products. These results suggest that confinement may lead to improving some eating behaviors for those students that already had good baseline habits [35]. This agrees with another work where an association between healthy behaviors was observed [36].

### 4.3. Changes in Diet and Dietary Patterns during the COVID-19 Pandemic Confinement according to the Perceived Socioeconomic Position

During the confinement, there was a higher risk of worsening their diet for adolescents from the most disadvantaged socioeconomic position tertile, compared to the ones from the middle and most advantaged tertiles (21% higher risk). This could also contribute to increasing socioeconomic inequalities in health and overweight/obesity, as pointed out by different studies [37].

We found an inverse relationship of the perceived socioeconomic position of adolescents with the reduction in the consumption of cereals, fruit, and vegetables and with the increase in the consumption of convenience foods. This is in line with studies that point out that the consumption of unhealthy foods related to obesity shows a socioeconomic pattern in Spanish children and adolescents [38]. These studies also report the strongest dietary inequalities in the consumption of fruit and vegetables, with lower socioeconomic groups having less possibilities to consume these types of food that are at the base of the Mediterranean Diet pyramid [39,40]. Besides, the decrease in the variety of foods and the possible loss of culinary skills are related to the increased consumption of convenience foods. Both of these facts deepen the gap with the Mediterranean pattern, not only as a healthy diet, but also as a model of sustainable and culturally adapted lifestyle [41].

Other recent studies with similar populations also show how socioeconomic factors and the educational level of parents play an important role in the daily consumption of fruit, vegetables, and white meat [42]. In this line, our findings suggest that adolescents with an advantaged socioeconomic position may have a healthier food environment at home. Instead, students with a disadvantaged position may benefit more from a healthy food environment at school [43]. Improving nutritional knowledge (school and family), establishing routines or rules related to meals, promoting home food availability of fruits and less healthful alternatives can be valuable ways of promoting healthy eating and reducing inequalities in dietary behaviors in adolescents [44]. Greater economic and educational resources can contribute to healthier dietary behavior through increased food budgets, nutritional knowledge, planning food purchases, and cooking skills [45,46,47,48]. Given that the availability and the purchase of food for the household are partly dependent on parents, these factors may play an important role in food choices by adolescents. Additionally, it must be noted that some factors can be influenced by organizational or community questions. Limited mobility during confinement in areas of greater socioeconomic deprivation may also lead to exposure to unhealthy food environments. This would increase dietary inequalities, food poverty, and malnutrition, as observed in other studies [34,49], because of the possible limitation in supply, both in terms of variety and price. Besides, data from the Catalan government [50] suggest that the rate of COVID-19 infection is higher in the most deprived areas of the region [51]. Thus, the prevalence and severity of the COVID-19 pandemic is magnified by the coexistence of previous chronic diseases (syndemic), which are also associated with social determinants of health [52].

These results highlight the increase in nutritional and health inequalities during the COVID-19 pandemic lockdown. They also suggest that governments and policy makers need to consider socioeconomic differences when planning policies. Indeed, it has been shown during the COVID-19 pandemic that incorporating equally severe policies (e.g., confinement for all people regardless of their social situation) in the whole society can contribute to widening social inequalities in health, since these policies usually have a greater negative impact on the most vulnerable population. Therefore, it could be interesting to disseminate easy and practical dietary recommendations for families to help them adapt their purchases and food intake to different situations. These recommendations should emphasize not only nutritional aspects, but also dietary, gastronomic, and culinary planning, and the importance of family meals as a space for socialization and communication in which parents act as role models for adolescents’ food preferences [53]. Furthermore, urban planning should consider accessibility and proximity to local markets or grocery stores with an abundance of fresh and seasonal products and invest in the development of the digital market for fresh and refrigerated products, taking into account the sustainable development goals [54].

### 4.4. Limitations

One potential limitation is that the data were self-reported, so there may be some recall bias or social desirability bias towards engaging in healthy behaviors or reporting inaccurate socioeconomic data. However, the use of self-reported questionnaires is a common method in this type of study, because of their low cost and easy administration. Moreover, the changes observed are short term, and it should be studied whether they would be maintained during longer periods of confinement. Additionally, the sample of students was not large, and the convenience sampling could have led to a bias related to the participation of students that were most motivated to engage in healthy behaviors. For this reason, the sample of our study may not be representative of the general adolescent population of Central Catalonia. However, despite these limitations, our study provides us with important information about changes in the diet of a cohort of adolescents during confinement. It also allows us to link such changes to pre-pandemic habits, and to do a follow up. Finally, our data suggest that these changes were unevenly distributed according to perceived socioeconomic position, increasing nutritional inequalities.

## 5. Conclusions

The results of this study show how confinement has led to changes in the dietary habits of adolescents. The main changes are represented by an increased consumption of fruit and a decreased consumption of soft drinks, sweets and pastries, and convenience foods. We also showed a more irregular pattern of meal distribution. These changes are related to the perceived socioeconomic position of the students, and the risk to worsen the diet is 21% higher in adolescents of the most disadvantaged socioeconomic position. These findings could help to develop future public policies and nutritional recommendations that must be adapted to the characteristics of the most vulnerable population groups, to avoid increasing nutritional and health inequalities. Likewise, knowing the eating behavior of adolescents in different situations can also contribute to improving interventions for the adoption of healthy diets and, thus, impact on the future health of the population.

## Figures and Tables

**Table 1:** Distribution of participants according to the independent variables studied. Subsample of the DESKcohort project collected during the confinement due to the COVID-19 pandemic.

Variable	*n*	%
**Gender**		
Girl	213	70.3
Boy	90	29.7
**Course**		
ISCED 2	169	55.8
ISCED 3	134	44.2
Perceived socioeconomic position * (mean, SD)	62.48	13.8
**Size of municipality**		
≤5000 inhabitants	87	28.7
5001–20,000 inhabitants	126	41.6
>20,000 inhabitants	79	26.1
No data available	11	3.6
**Body mass index**		
Underweight or normal weight **	250	82.5
Overweight or obesity	50	16.5
No data	3	1.0
**Physical activity**		
Compliance with WHO recommendations	107	35.3
Under WHO recommendations	181	59.7
No data	15	5.0
**Self-perceived health status**		
Excellent or very good	163	53.8
Good, fair, or poor	140	46.2
**Sleep quality**		
Very good or good	184	60.7
Very poor or poor	119	39.3

* In the perceived socioeconomic position variable, the heading “*n*” is substituted for “mean” and “%” for “SD”, ** underweight and normal weight participants were grouped together, as only 7 participants were classified as underweight. Abbreviations: ISCED: UNESCO International Standard Classification of Education; SD: standard deviation; WHO: World Health Organization.

**Table 2:** Proportion of people showing changes in the intake of different types of food among high-school students from Central Catalonia during the COVID-19 confinement. Results represented by tertiles of the Spanish Health Eating Index (HEI).

	Total (*n* = 303)								
	Decrease			No Changes			Increase		
	*n*	%	95% CI	*n*	%	95% CI	*n*	%	95% CI
Highest tertile Spanish HEI (*n* = 100)									
Consumption of cereals	12	12.0	(6.9–20.0)	70	70.0	(60.3–78.2)	18	18.0	(11.6–26.8)
Consumption of legumes	15	15.0	(9.2–23.4)	73	73.0	(63.4–80.8)	12	12.0	(6.9–20.0)
Consumption of fruit	12	12.0	(6.9–20.0)	46	46.0	(36.5–55.8)	42	42.0	(32.7–51.9)
Consumption of vegetables	3	3.0	(1.0–8.9)	83	83.0	(74.3–89.2)	14	14.0	(8.5–22.3)
Consumption of dairy products	23	23.0	(15.8–32.3)	56	56.0	(46.1–65.4)	21	21.0	(14.1–30.1)
Consumption of meat	16	16.0	(10.0–24.6)	74	74.0	(64.5–81.7)	10	10.0	(5.5–17.6)
Consumption of fish	21	21.0	(14.1–30.1)	72	72.0	(62.4–79.9)	7	7.0	(3.4–14.0)
Consumption of eggs	14	14.0	(8.5–22.3)	71	71.0	(61.3–79.1)	15	15.0	(9.2–23.4)
Consumption of convenience food	50	50.0	(40.3–59.7)	42	42.0	(32.7–51.9)	8	8.0	(4.0–15.2)
Consumption of sweets	39	39.0	(29.9–48.9)	34	34.0	(25.4–43.8)	27	27.0	(19.2–36.6)
Consumption of soft drinks	44	44.0	(34.6–53.9)	44	44.0	(34.6–53.9)	12	12.0	(6.9–20.0)
Intermediate tertile Spanish HEI (*n* = 98)									
Consumption of cereals	8	8.2	(4.1–15.5)	69	70.4	(60.6–78.6)	21	21.4	(14.4–30.7)
Consumption of legumes	16	16.3	(10.2–25.0)	67	68.4	(58.5–76.8)	15	15.3	(9.4–23.9)
Consumption of fruit	14	14.3	(8.6–22.7)	46	46.9	(37.3–56.8)	38	38.8	(29.6–48.8)
Consumption of vegetables	13	13.3	(7.8–21.6)	57	58.2	(48.2–67.5)	28	28.6	(20.5–38.3)
Consumption of dairy products	20	20.4	(13.5–29.6)	57	58.2	(48.2–67.5)	21	21.4	(14.4–30.7)
Consumption of meat	21	21.4	(14.4–30.7)	62	63.3	(53.3–72.2)	15	15.3	(9.4–23.9)
Consumption of fish	28	28.6	(20.5–38.3)	56	57.1	(47.2–66.6)	14	14.3	(8.6–22.7)
Consumption of eggs	10	10.2	(5.6–18.0)	71	72.4	(62.8–80.4)	17	17.3	(11.0–26.2)
Consumption of convenience food	51	52.0	(42.2–61.8)	39	39.8	(30.6–49.8)	8	8.2	(4.1–15.5)
Consumption of sweets	38	38.8	(29.6–48.8)	32	32.7	(24.1–42.6)	28	28.6	(20.5–38.3)
Consumption of soft drinks	38	38.8	(29.6–48.8)	43	43.9	(34.4–53.9)	17	17.3	(11.0–26.2)
Lowest tertile Spanish HEI (*n* = 105)									
Consumption of cereals	9	8.6	(4.5–15.7)	74	70.5	(61.0–78.4)	22	21.0	(14.2–29.8)
Consumption of legumes	26	24.8	(17.4–33.9)	66	62.9	(53.2–71.6)	13	12.4	(7.3–20.2)
Consumption of fruit	22	21.0	(14.2–29.8)	45	42.9	(33.7–52.5)	38	36.2	(27.6–45.8)
Consumption of vegetables	14	13.3	(8.0–21.3)	61	58.1	(48.4–67.2)	30	28.6	(20.7–38.0)
Consumption of dairy products	14	13.3	(8.0–21.3)	71	67.6	(58.1–75.9)	20	19.0	(12.6–27.7)
Consumption of meat	9	8.6	(4.5–15.7)	73	69.5	(60.0–77.6)	23	21.9	(15.0–30.9)
Consumption of fish	27	25.7	(18.2–34.9)	60	57.1	(47.5–66.3)	18	17.1	(11.1–25.6)
Consumption of eggs	14	13.3	(8.0–21.3)	73	69.5	(60.0–77.6)	18	17.1	(11.1–25.6)
Consumption of convenience food	40	38.1	(29.3–47.8)	50	47.6	(38.2–57.2)	15	14.3	(8.8–22.4)
Consumption of sweets	36	34.3	(25.8–43.9)	35	33.3	(25.0–42.9)	34	32.4	(24.1–41.9)
Consumption of soft drinks	31	29.5	(21.6–39.0)	47	44.8	(35.5–54.4)	27	25.7	(18.2–34.9)

**Table 3:** Proportion of people showing changes in the different eating behaviors among high-school students from Central Catalonia during the COVID-19 confinement. Results represented by tertiles of the Spanish Health Eating Index (HEI).

	Total (*n* = 303)								
	Decrease			No Changes			Increase		
	*n*	%	95% CI	*n*	%	95% CI	*n*	%	95% CI
Highest tertile Spanish HEI (*n* = 100)									
Amount of food consumed	21	21.0	(14.1–30.1)	50	50.0	(40.3–59.7)	29	29.0	(20.9–38.7)
Variety of foods consumed	13	13.0	(7.7–21.2)	67	67.0	(57.2–75.5)	20	20.0	(13.3–29.0)
Regularity of meal hours	33	33.0	(24.5–42.8)	49	49.0	(39.3–58.8)	18	18.0	(11.6–26.8)
Meals skipped	23	23.0	(15.8–32.3)	50	50.0	(40.3–59.7)	27	27.0	(19.2–36.6)
Consumption of snacks between meals	18	18.0	(11.6–26.8)	27	27.0	(19.2–36.6)	55	55.0	(45.1–64.5)
Consumption of fresh food	18	18.0	(11.6–26.8)	57	57.0	(47.1–66.4)	25	25.0	(17.5–34.4)
Consumption of packaged food	22	22.0	(14.9–31.2)	57	57.0	(47.1–66.4)	21	21.0	(14.1–30.1)
Intermediate tertile Spanish HEI (*n* = 98)									
Amount of food consumed	23	23.5	(16.1–32.9)	46	46.9	(37.3–56.8)	29	29.6	(21.4–39.4)
Variety of foods consumed	17	17.3	(11.0–26.2)	64	65.3	(55.4–74.1)	17	17.3	(11.0–26.2)
Regularity of meal hours	42	42.9	(33.4–52.8)	39	39.8	(30.6–49.8)	17	17.3	(11.0–26.2)
Meals skipped	24	24.5	(17.0–34.0)	47	48.0	(38.2–57.8)	27	27.6	(19.6–37.2)
Consumption of snacks between meals	11	11.2	(6.3–19.2)	25	25.5	(17.8–35.1)	62	63.3	(53.3–72.2)
Consumption of fresh food	17	17.3	(11.0–26.2)	51	52.0	(42.2–61.8)	30	30.6	(22.3–40.4)
Consumption of packaged food	22	22.4	(15.2–31.8)	55	56.1	(46.1–65.6)	21	21.4	(14.4–30.7)
Lowest tertile Spanish HEI (*n* = 105)									
Amount of food consumed	24	22.9	(15.8–31.9)	43	41.0	(31.9–50.6)	38	36.2	(27.6–45.8)
Variety of foods consumed	21	20.0	(13.4–28.8)	56	53.3	(43.7–62.7)	28	26.7	(19.1–36.0)
Regularity of meal hours	46	43.8	(34.6–53.4)	39	37.1	(28.4–46.8)	20	19.0	(12.6–27.7)
Meals skipped	21	20.0	(13.4–28.8)	52	49.5	(40.1–59.0)	32	30.5	(22.4–40.0)
Consumption of snacks between meals	22	21.0	(14.2–29.8)	29	27.6	(19.9–37.0)	54	51.4	(41.9–60.9)
Consumption of fresh food	25	23.8	(16.6–32.9)	56	53.3	(43.7–62.7)	24	22.9	(15.8–31.9)
Consumption of packaged food	31	29.5	(21.6–39.0)	50	47.6	(38.2–57.2)	24	22.9	(15.8–31.9)

## Data Availability

The data presented in this study are available upon reasonable request to the corresponding author. The data are not publicly available due to confidentiality reasons.

## Supplementary Materials

The following are available online at https://www.mdpi.com/article/10.3390/nu13051577/s1, Figure S1: Changes in food consumption and eating behavior among high-school students in Central Catalonia during the confinement due to the COVID-19 pandemic. DESK-COVID project, 2020, Figure S2: Relationship between less healthy eating variables during the COVID-19 confinement and a more disadvantaged socioeconomic position of the high-school students from Central Catalonia. DESK-COVID project, 2020.
