# Supplementary material for: Social Inequalities in Changes in Diet in Adolescents during Confinement Due to COVID-19 in Spain: The DESKcohort Project
Source: Nutrients. 2021 May 8;13(5):1577. doi: 10.3390/nu13051577 (PMC8151229; doi:10.3390/nu13051577)
Supplement: Supplementary file 1 [file nutrients-13-01577-s001.zip › nutrients-1196536-SI.pdf]

Figure S1. Changes in food consumption and eating behavior among high-school students in Central Catalonia during the confinement due to the COVID-19 pandemic. DESK-COVID project, 2020.

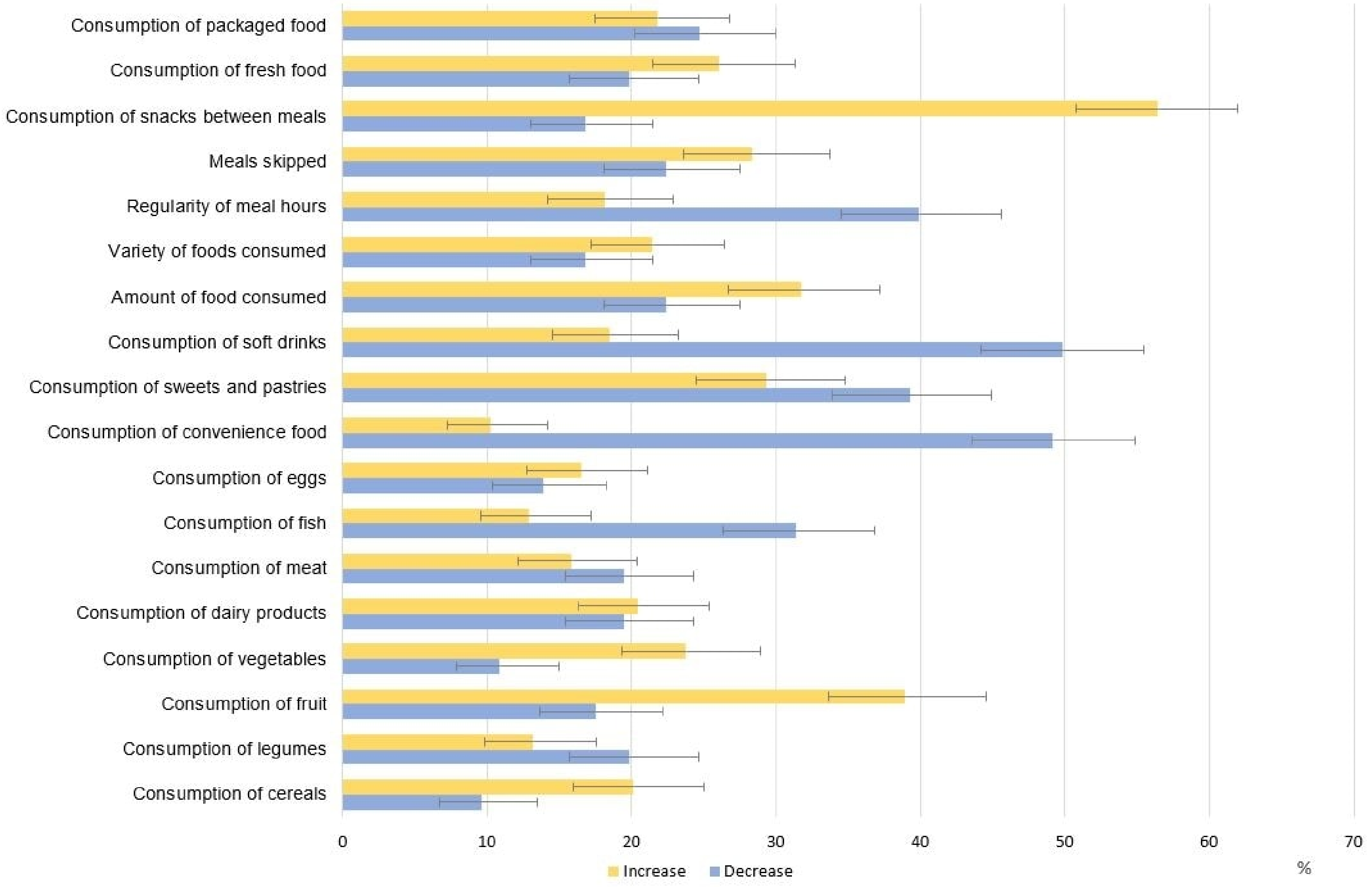

Figure S2. Relationship between less healthy eating variables during the COVID-19 confinement and a more disadvantaged socioeconomic position of the high-school students from Central Catalonia. DESK-COVID project, 2020.

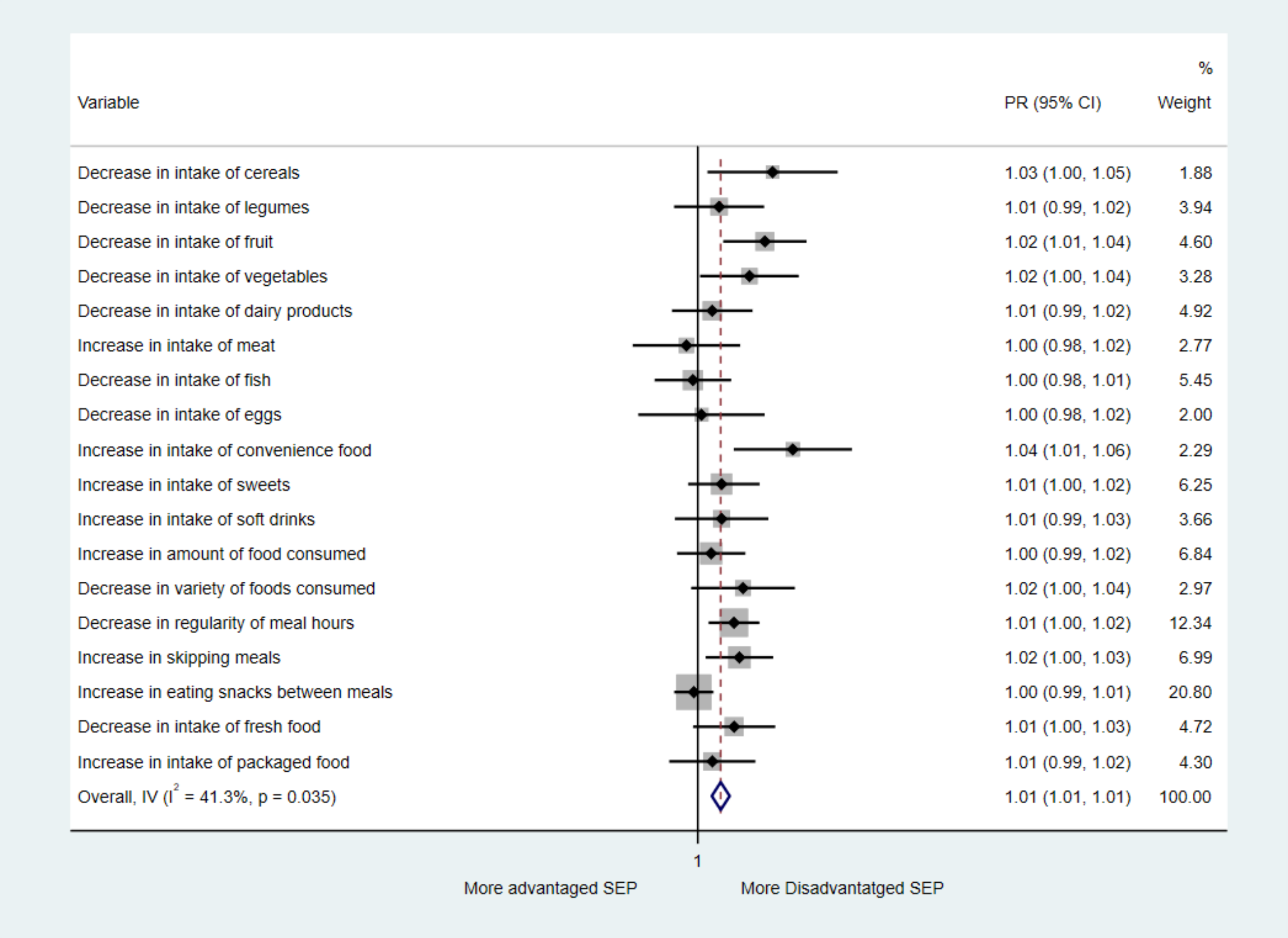

Perceived socioeconomic position has been considered as a continuous variable. Prevalence ratio has been adjusted by sex, age, and self-perceived health status. Abbreviations: PR: Prevalence Ratio; SEP: Socioeconomic Position; IV: Independent Variable; I2: I-squared (proportion of total variation in effect estimate due to between-study heterogeneity).
